# Preoperative C-reactive protein to albumin ratio predicts anastomotic leakage after esophagectomy for thoracic esophageal cancer: a single-center retrospective cohort study

**DOI:** 10.1186/s12893-021-01344-7

**Published:** 2021-09-21

**Authors:** Atsushi Sugimoto, Takahiro Toyokawa, Yuichiro Miki, Mami Yoshii, Tatsuro Tamura, Katsunobu Sakurai, Naoshi Kubo, Hiroaki Tanaka, Shigeru Lee, Kazuya Muguruma, Masakazu Yashiro, Masaichi Ohira

**Affiliations:** 1 Department of Gastroenterological Surgery, Osaka City University Graduate School of Medicine, 1-4-3 Asahimachi, Abeno-ku, Osaka, 545-8585 Japan; 2 Department of Gastroenterological Surgery, Osaka City General Hospital, 2-13-22 Miyakojimahondori, Miyakojima-ku, Osaka, 534-0021 Japan

**Keywords:** C-reactive protein-to-albumin ratio, Anastomotic leakage, Esophageal cancer

## Abstract

**Background:**

Postoperative anastomotic leakage (AL) is associated with not only prolonged hospital stay and increased medical costs, but also poor prognosis in esophageal cancer. Several studies have addressed the utility of various inflammation-based and/or nutritional markers as predictors for postoperative complications. However, none have been documented as specific predictors for AL in esophageal cancer. We aimed to identify predictors of AL after esophagectomy for thoracic esophageal cancer, focusing on preoperative inflammation-based and/or nutritional markers.

**Methods:**

We retrospectively analyzed 295 patients who underwent radical esophagectomy for thoracic esophageal squamous cell carcinoma between June 2007 and July 2020. As inflammation-based and/or nutritional markers, Onodera prognostic nutritional index, C-reactive protein (CRP)-to-albumin ratio (CAR) and modified Glasgow prognostic score were investigated. Optimal cut-off values of inflammation-based and/or nutritional markers for AL were determined by receiver operating characteristic curves. Predictors for AL were analyzed by logistic regression modeling.

**Results:**

AL was observed in 34 patients (11.5%). In univariate analyses, preoperative body mass index (≥ 22.1 kg/m^2^), serum albumin level (≤ 3.8 g/dL), serum CRP level (≥ 0.06 mg/dL), CAR (≥ 0.0139), operation time (> 565 min) and blood loss (≥ 480 mL) were identified as predictors of AL. Multivariate analyses revealed higher preoperative CAR (≥ 0.0139) as an independent predictor of AL (*p* = 0.048, odds ratio = 3.02, 95% confidence interval 1.01–9.06).

**Conclusion:**

Preoperative CAR may provide a useful predictor of AL after esophagectomy for thoracic esophageal squamous cell carcinoma.

## Background

Anastomotic leakage (AL) is one of the most serious complications after esophagectomy. Postoperative AL is reportedly associated with not only prolonged postoperative hospital stays and increased medical costs, but also poor prognosis for esophageal cancer [1]. The incidence of AL reportedly shows a wide range of 0–35% after esophagectomy [2]. The Japanese National Clinical Database (NCD) of digestive surgery demonstrated an incidence of 12.6% for AL after esophagectomy for esophageal cancer [3]. Although perioperative management and surgical techniques have been improved, the incidence of AL after esophagectomy for esophageal cancer remains unsatisfactory. To reduce AL after esophagectomy, identifying preoperative and surgical predictors of AL is important.

Serum albumin has been used as a simple nutritional marker for predicting postoperative complications in various gastrointestinal surgeries [4]. Serum C-reactive protein (CRP) has been used as a simple marker of systemic inflammation predicting postoperative infectious complications [5]. Several recent studies have reported the utility of various inflammation-based and/or nutritional markers as predictors of postoperative complications and poor prognosis in esophageal cancer, such as the Onodera’s prognostic nutritional index (PNI), CRP-to-albumin ratio (CAR) and modified Glasgow prognostic score (mGPS), which are calculated based on laboratory data including serum albumin or CRP [6–8]. Some studies have reported preoperative PNI, CAR and mGPS as predictors for AL after gastrointestinal cancer surgery [9–11]. However, no studies appear to have examined the association between preoperative inflammation-based and/or nutritional markers and AL after esophageal cancer surgery. The significance of inflammation-based and/or nutritional markers and the optimal cut-off values to predict AL using these markers have yet to be clarified.

This study therefore aimed to identify specific predictors of AL after radical esophagectomy for thoracic esophageal squamous cell carcinoma, focusing on preoperative inflammation-based and/or nutritional markers, such as albumin, CRP, PNI, CAR, and mGPS.

## Patients and methods

### Patients

We retrospectively analyzed consecutive patients who underwent subtotal esophagectomy with two- or three-field lymphadenectomy and reconstruction using a gastric tube by cervical anastomosis for thoracic esophageal squamous cell carcinoma in the Department of Gastroenterological Surgery at Osaka City University Hospital between June 2007 and July 2020. Patients with clinical stage IVB, R2 resection, synchronous surgery for other cancers, combined resection of other organs, two-stage reconstruction, ante-thoracic route reconstruction, or mediastinoscopy-assisted esophagectomy were excluded. The ethics committee at our institution approved this retrospective study of clinical data, which was conducted in accordance with the principles of the Declaration of Helsinki.

All clinical and surgical data were collected from electronic medical records as follows: age, sex, body mass index (BMI), habitual tobacco use, habitual alcohol use, comorbidities according to the Charlson comorbidity index (CCI) [12], serum albumin level, serum CRP level, PNI, CAR, mGPS, the American Society of Anesthesiologists classification of physical status (ASA-PS), tumor location, clinical T stage, clinical N stage, clinical stage, preoperative treatment (chemotherapy, chemoradiotherapy or none), thoracic procedure (open or minimally invasive esophagectomy: MIE), abdominal procedure (open or hand-assisted laparoscopic surgery: HALS), lymph node dissection (three-field or two-field), anastomosis method (hand-sewn or mechanical anastomosis), reconstruction route (retrosternal or posterior mediastinal), operation time, blood loss, and the timing of surgery (2007–2014 or 2015–2020). Clinical stage was determined based on the 8th edition of the Union for International Cancer Control TNM classification of malignant tumors [13]. Preoperative inflammation-based and/or nutritional markers were calculated as shown in Table 1. Laboratory data were obtained within 1 week before the operation.

**Table 1:** Definitions of preoperative inflammation-based and/or nutritional markers

Onodera prognostic nutritional index (PNI)	
10 × albumin (g/dL) + 0.005 × TLC (/μL)	
C-reactive protein (CRP)-to-albumin ratio (CAR)	
CRP / albumin (g/dL)	
Modified Glasgow prognostic score (mGPS)	Score
CRP ≤ 0.5 mg/dL and albumin ≥ 3.5 g/dL	0
CRP > 0.5 mg/dL or albumin < 3.5 g/dL	1
CRP > 0.5 mg/dL and albumin < 3.5 g/dL	2

*TLC* total lymphocytes counts, *CRP* serum C-reactive protein

### Preoperative treatment

All the patients had various radiological tests for preoperative diagnosis and staging, such as upper gastrointestinal endoscopy, esophagography, contrast-enhanced computed tomography (CT) between the neck and upper abdomen, and positron emission tomography (PET) CT if necessary. Preoperative chemoradiotherapy was performed for patients with clinical T4a disease with chemotherapy (5-fluorouracil [5-FU]/nedaplatin) and radiotherapy (total dose, 41.4 Gy) until March 2012. Additional surgery was performed if the tumor was still present and considered likely to be resectable. From April 2012, neoadjuvant chemotherapy (NAC) was performed for patients with clinical stage II/III disease. The regimens of neoadjuvant chemotherapy included 5-FU/nedaplatin, 5-FU/cisplatin and 5-FU/cisplatin/docetaxel.

### Surgical procedure and anastomotic technique

All patients underwent an open esophagectomy or MIE which included video-assisted transthoracic surgery and robotic-assisted surgery, accompanied by open surgery or HALS for mobilizing the stomach. The gastric tube was pulled up through the retrosternal or posterior mediastinal route. Three-field (cervical, mediastinal, and abdominal field) lymph node dissection was mainly performed, with two-field (mediastinal and abdominal fields) lymph node dissection applied only for non-advanced lower thoracic esophageal cancer. In terms of the reconstruction route, the posterior mediastinal route was preferred until March 2015, after which a retrosternal route was preferred. For the cervical anastomotic technique, mechanical end-to-side anastomosis with a circular stapler was mainly performed until March 2015, after which hand-sewn end-to-end anastomosis with monofilament absorbable sutures was performed.

### Definition of AL

When clinical signs suggestive of AL (redness, swelling and tenderness of the neck, re-elevation of white blood cell count and C-reactive protein after oral intake, and fever) were observed, oral contrast esophagography and CT were performed to confirm the AL. AL was defined as a full-thickness gastrointestinal defect involving the esophagus, anastomosis, staple line, or conduit, irrespective of presentation or method of identification, according to the Esophagectomy Complications Consensus Group (ECCG) consensus definitions [14]. Severity of AL was evaluated according to the Clavien-Dindo classification (CDC) [15]. Patients with AL were allocated to the AL group and patients without AL were allocated to the no anastomotic leakage (NAL) group.

### Determination of cut-offs

Cut-off values for age, BMI, CCI, albumin, CRP, PNI, CAR, mGPS, operation time and blood loss were determined at the point of maximal Youden index based on the receiver operating characteristic (ROC) curve. All patients were classified into one of these two groups based on the cut-off values.

### Statistical analysis

Statistical comparisons of baseline data were performed using the Mann–Whitney *U* test for continuous variables, and the chi-squared test or Fisher's exact test for categorical variables. To identify factors predictive of AL after esophagectomy, multiple logistic regression analysis was performed. Odds ratios (ORs) and 95% confidence intervals (95% CIs) were calculated. Separate multivariate analyses were performed to compare the predictive values of albumin, CRP and individual inflammation-based and/or nutritional markers showing values of *p* < 0.1 in univariate analyses because PNI, CAR and mGPS include albumin and CRP level in the estimations. Values of *p* < 0.05 were considered statistically significant. All data analysis was conducted using JMP® version 13 (SAS Institute Inc., Cary, NC, USA).

## Results

### Patients characteristics

A total of 295 patients (241 male, 54 female) were included in this study. Median age, BMI, operation time and estimated blood loss were 65 years (interquartile range [IQR], 59–71 years), 21.2 kg/m^2^ (IQR, 19.5–23.8 kg/m^2^), 561 min (IQR, 509–613 min), 300 ml (IQR, 190–550 ml), respectively. Postoperative AL was observed in 34 patients (11.5%). Among the patients with AL, 2 patients were categorized to CDC class I, 6 patients to CDC class II, 23 patients to CDC class IIIa, and 3 patients to CDC class IIIb, respectively. There were no cases of postoperative death due to AL. Table 2 shows the association between clinicopathological characteristics and AL.

**Table 2:** Association between clinicopathological factors and patients with or without anastomotic leakage

		All case	Group NAL	Group AL	
		N = 295	N = 261	N = 34	p value
Age	(Years, IQR)	65 (59–71)	66 (59–71)	65 (59–71)	0.845
	≥ 53	265 (89.8%)	232 (88.9%)	33 (97.1%)	0.224*
	< 53	30 (10.2%)	29 (11.1%)	1 (2.9%)	
Gender	Male	241 (81.7%)	211 (80.8%)	30 (88.2%)	0.355*
	Female	54 (18.3%)	50 (19.2%)	4 (11.8%)	
BMI	(kg/m2, IQR)	21.2 (19.4–23.5)	21.0 (19.4–23.5)	22.2 (19.8–23.8)	0.433
	≥ 22.1	114 (38.6%)	95 (36.4%)	19 (55.9%)	0.028
	< 22.1	181 (61.4%)	166 (63.6%)	15 (44.1%)	
Habitual tobacco use	Yes	235 (79.7%)	204 (78.2%)	31 (91.2%)	0.11*
	No	60 (20.3%)	57 (21.8%)	3 (8.8%)	
Habitual alcohol use	Yes	235 (79.7%)	205 (78.5%)	30 (88.2%)	0.257*
	No	60 (20.3%)	56 (21.5%)	4 (11.8%)	
Steroid use	Yes	4 (1.4%)	3 (1.2%)	1 (2.9%)	0.389*
	No	291 (98.6%)	258 (98.9%)	33 (97.1%)	
CCI	(IQR)	0 (0–1)	0 (0–1)	0 (0–1)	0.829
	≤ 2	283 (95.9%)	252 (96.6%)	31 (91.2%)	0.149
	≥ 3	12 (4.1%)	9 (3.5%)	3 (8.8%)	
Albumin	(g/dL, IQR)	3.9 (3.7–4.2)	3.9 (3.7–4.2)	3.8 (3.6–4.1)	0.203
	> 3.8	172 (58.3%)	157 (60.2%)	15 (44.1%)	0.075
	≤ 3.8	123 (41.7%)	104 (39.8%)	19 (55.9%)	
CRP	(mg/dL, IQR)	0.1 (0.04–0.3)	0.10 (0.04–0.28)	0.14 (0.06–0.43)	0.075
	≥ 0.06	200 (67.8%)	171 (65.5%)	29 (85.3%)	0.02
	< 0.06	95 (32.2%)	90 (34.5%)	5 (14.7%)	
PNI	(IQR)	47.4 (44.4–50.7)	47.4 (44.4–50.7)	48.0 (44.3–50.6)	0.82
	≥ 42.5	258 (87.5%)	226 (86.6%)	32 (94.1%)	0.279*
	< 42.5	37 (12.5%)	35 (13.4%)	2 (5.9%)	
CAR	(IQR)	0.024 (0.01–0.08)	0.024 (0.009–0.073)	0.037 (0.015–0.123)	0.072
	≥ 0.0139	200 (67.8%)	170 (65.1%)	30 (88.2%)	0.007
	< 0.0139	95 (32.2%)	91 (34.9%)	4 (11.8%)	
mGPS	0	235 (79.7%)	210 (80.5%)	25 (73.5%)	0.345
	1, 2	60 (20.3%)	51 (19.5%)	9 (26.5%)	
ASA-PS	1, 2	275 (93.2%)	245 (93.9%)	30 (88.2%)	0.265*
	3	20 (6.8%)	16 (6.1%)	4 (11.8%)	
Location of tumor	Ut	53 (18.0%)	47 (18.0%)	6 (17.6%)	0.887
	Mt	172 (58.3%)	151 (57.9%)	21 (61.8%)	
	Lt	70 (23.7%)	63 (24.1%)	7 (20.6%)	
cT (UICC8)	1, 2	161 (54.6%)	140 (53.6%)	21 (61.8%)	0.371
	3, 4	134 (45.4%)	121 (46.4%)	13 (38.4%)	
cN (UICC8)	Yes	162 (54.9%)	144 (55.2%)	18 (52.9%)	0.806
	No	133 (45.1%)	117 (44.8%)	16 (47.1%)	
cStage (UICC8)	I, II	157 (53.2%)	138 (52.9%)	19 (55.9%)	0.741
	III, IV	138 (46.8%)	123 (47.1%)	15 (44.1%)	
Preoperative treatment	Chemotherapy	154 (52.2%)	132 (50.6%)	22 (64.7%)	0.158
	Chemoradiotherapy	16 (5.4%)	16 (6.1%)	0 (0.0%)	
	Surgical only	125 (43.4%)	113 (43.3%)	12 (35.3%)	

*Fisher's exact test

*IQR* interquartile range, *BMI* body mass index, *CCI* Charlson comorbidity index, *CRP* serum C-reactive protein;

*PNI* Onodera’s prognostic nutritional index, *CAR* C-reactive protein to albumin ratio, *mGPS* modified Glasgow prognostic score

*ASA-PS* the American Society of Anesthesiologists classification of physical status

*UICC* the Union for International Cancer Control

### ROC curve analyses

**Fig. 1:**
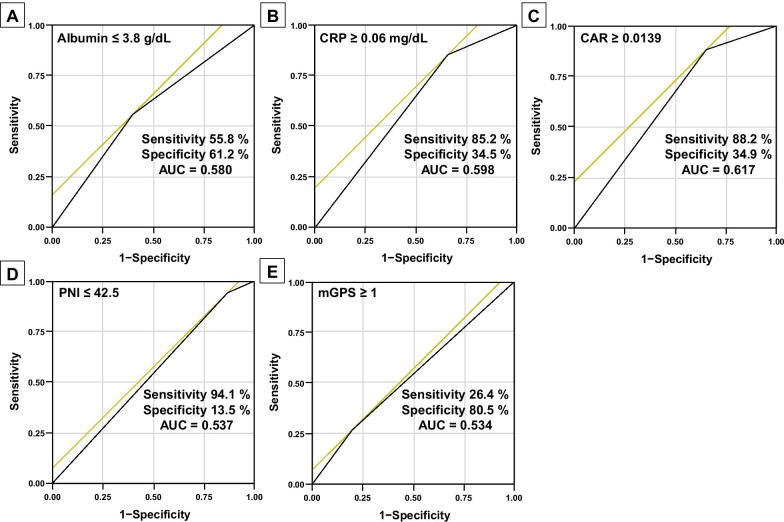
ROC curve-analyses predicting anastomotic leakage after esophagectomy for albumin (**A**), C-reactive protein (CRP) (**B**), CRP-to-albumin ratio (CAR) (**C**), Onodera prognostic nutritional index (PNI) (**D**) and modified Glasgow prognostic score (mGPS) (**E**)

In the present study, values of 53 years for age, 22.1 kg/m^2^ for BMI, 3 for CCI, 3.8 g/dL for albumin, 0.06 mg/dL for CRP, 42.5 for PNI, 0.0139 for CAR, 1 for mGPS, 565 min for operation time, and 480 ml for blood loss provided the maximal Youden indexes and were used as cut-off values. When patients were classified into two groups based on these cut-off values, areas under the curve (AUCs) predicting AL after esophagectomy were 0.580 for albumin, 0.598 for CRP, 0.617 for CAR, 0.537 for PNI and 0.534 for mGPS, respectively (Fig. 1).

### Uni- and multivariate analyses of preoperative and surgical factors for AL

Among preoperative factors, AL was significantly associated with higher BMI (*p* = 0.028), and higher CRP level (*p* = 0.02) (Table 2). Among inflammation-based and/or nutritional markers, AL was significantly associated with preoperative higher CAR (*p* = 0.007) (Table 2). Among surgical factors, AL was significantly associated with longer operation time (*p* = 0.008) and greater blood loss (*p* = 0.036) (Table 3). Multivariate analysis that included variables showing values of *p* < 0.1 on univariate analyses revealed preoperative higher CAR, but not lower albumin level or higher CRP level, as an independent predictive factor for AL (*p* = 0.048, OR = 3.02, 95% CI 1.01–9.06) (Table 4).

**Table 3:** Associations between surgical factors and patients with or without anastomotic leakage

		All case	Group NAL	Group AL	
		N = 295	N = 261	N = 34	p value
Thoracic procedure	MIE	220 (74.6%)	192 (75.6%)	28 (82.4%)	0.268
	Open	75 (25.4%)	69 (26.4%)	6 (17.6%)	
Abdominal procedure	HALS	277 (93.9%)	246 (94.3%)	31 (91.2%)	0.447*
	Open	18 (6.1%)	15 (5.7%)	3 (8.8%)	
Lymoph node dissection	Three-field	278 (94.3%)	248 (95.0%)	30 (88.2%)	0.118*
	Two-field	17 (5.8%)	13 (5.0%)	4 (11.8%)	
Operation time	(minutes, IQR)	561 (509–613)	556 (506–611)	598 (550–636)	0.02
	> 565	137 (46.4%)	114 (43.7%)	23 (67.7%)	0.008
	≤ 565	158 (53.6%)	147 (56.3%)	11 (32.4%)	
Blood loss	(mL, IQR)	300 (190–550)	275 (180–540)	335 (223–743)	0.078
	≥ 480	85 (28.8%)	70 (26.8%)	15 (44.1%)	0.036
	< 480	210 (71.2%)	191 (73.2%)	19 (55.9%)	
Anastomosis method	Hand-sewn	160 (54.2%)	144 (55.2%)	16 (47.1%)	0.372
	Mechanical	135 (45.8%)	117 (44.8%)	18 (52.9%)	
Reconstruction route	Retrosternal	178 (60.3%)	157 (60.2%)	21 (61.8%)	0.856
	Posterior mediastinal	117 (39.7%)	104 (39.8%)	13 (38.4%)	
Residual tumor	R0	285 (96.6%)	251 (96.2%)	34 (100%)	0.612*
	R1	10 (3.4%)	10 (3.8%)	0 (0.0%)	
Timing of surgery	2007–2014	125 (42.4%)	110 (42.2%)	15 (44.1%)	0.827
	2015–2020	170 (57.6%)	151 (57.9%)	19 (55.9%)	

*Fisher's exact test

*IQR* interquartile range;

*MIE* minimally invasive esophagectomy, *HALS* hand-assisted laparoscopic surgery

**Table 4:** Uni- and multivariate analyses of preoperative and surgical factors for anastomotic leakage

Variables	Analysis with CAR		Analysis with albumin		Analysis with CRP	
	HR (95% CI)	*p* value	HR (95% CI)	*p* value	HR (95% CI)	*p* value
BMI ≥ 22.1 kg/m^2^ (vs < 22.1 kg/m^2^)	1.69 (0.80–3.56)	0.17	1.84 (0.88–3.88)	0.107	1.74 (0.82–3.66)	0.147
Operation time > 565 min (vs ≤ 565 min)	1.95 (0.87–4.37)	0.106	2.06 (0.92–4.59)	0.079	1.96 (0.87–4.41)	0.102
Blood loss ≥ 480 mL (vs < 480 mL)	1.42 (0.65–3.11)	0.226	1.52 (0.70–3.31)	0.231	1.45 (0.66–3.17)	0.35
CAR ≥ 0.0139 (vs < 0.0139)	3.02 (1.01–9.06)	0.048				
Albumin ≤ 3.8 g/dL (vs > 3.8 g/dL)			1.69 (0.81–3.54)	0.162		
CRP ≥ 0.06 mg/dL (vs < 0.06 mg/dL)					2.26 (0.82–6.21)	0.113

BMI, body mass index; CAR, C-reactive protein-to-albumin ratio; CRP: C-reactive protein;

OR, odds ratio; 95%CI, 95% confidence interval

## Discussion

The present study evaluated preoperative and surgical factors that correlated with AL in 295 patients who underwent esophagectomy and cervical anastomosis for thoracic esophageal squamous cell carcinoma. We identified high preoperative CAR (≥ 0.0139) as an independent predictor for AL. To the best of our knowledge, this is the first study to compare the impact of inflammation-based and nutritional markers for AL and to identify preoperative CAR as an independent predictor for AL after esophagectomy. Preoperative CAR may represent a useful marker to predict AL after esophagectomy for thoracic esophageal squamous cell carcinoma.

Several studies have reported that preoperative inflammation-based and/or nutritional markers are associated with postoperative complications in various gastrointestinal cancer surgeries [16]. On the other hand, only a few reports have indicated associations between preoperative inflammation-based and/or nutritional markers and AL. Yu et al. demonstrated CAR was an independent risk factor for AL in elderly patients after curative colorectal surgery [10]. Oshi et al. reported PNI as an independent risk factor for AL after laparoscopic total gastrectomy [9]. However, associations between preoperative inflammation-based and/or nutritional markers and AL have not been reported for esophageal cancer surgery, so the best markers to predict AL remain unclear. The present study compared the predictive ability of CAR, PNI and mGPS for AL, and identified high preoperative CAR (≥ 0.0139), but not PNI or mGPS, as an independent predictor of AL after esophagectomy. CAR showed a greater AUC than PNI or mGPS for the prediction of AL. Our findings suggest preoperative CAR as a promising predictive marker for AL of cervical anastomosis after esophagectomy for thoracic esophageal cancer.

Albumin has been used as a simple nutritional marker in gastrointestinal surgeries [4, 17]. Preoperative hypoalbuminemia has been reported as a risk factor for AL in esophageal cancer [18]. CRP has been used as a simple marker of systemic inflammation. Previous studies have reported higher preoperative CRP level to be associated with postoperative infectious complications following gastrointestinal cancer surgery [5, 19]. However, no association between preoperative CRP and AL has been reported. In the present study, although univariate analysis revealed higher CAR, higher CRP and lower albumin levels as significantly associated with AL, multivariate analyses identified only higher CAR as an independent predictor for AL. Furthermore, ROC curve analyses revealed that the AUC of preoperative CAR for predicting AL was higher than those of preoperative albumin or CRP level alone. Similar to our findings, Ge et al. demonstrated that CAR offered higher diagnostic accuracy than CRP alone for postoperative complications in colorectal surgery [20]. Our findings suggest that CAR calculated by serum CRP and albumin level has superior predictive value for AL after esophagectomy than serum albumin or CRP level alone.

The exact explanation underlying the association between preoperative CAR and the development of AL after esophagectomy for esophageal cancer is unclear. Patients with esophageal cancer are at risk of malnutrition and systemic inflammation due to several factors, such as cancer-induced higher metabolism, reduced dietary intake, protein turnover and cachexia [21]. Albumin has a long half-life as a body protein, so hypoalbuminemia reflects prolonged malnutrition [22]. Hypoalbuminemia has been associated with poor tissue healing and reduction of tensile strength in anastomoses due to decreased collagen synthesis at the anastomotic site or surgical wound [23]. On the other hand, prolonged inflammation can impair collagen synthesis and induce anastomotic dehiscence [24]. CRP is primarily synthesized in the liver as an acute-phase response protein, and CRP levels correlate with severity of inflammation [25]. Tumor cells produce inflammatory cytokines, such as tumor necrosis factor α and interleukin (IL)-1 and -6, and IL-6 is a major stimulator inducing CRP synthesis [26]. Increased serum IL-6 levels have been reported as a predictor of AL in gastrointestinal surgery [27]. The preoperative nutritional and inflammatory status represented by CAR may thus be responsible for the development of AL.

Improving preoperative systemic inflammation and nutritional status could represent a potential strategy to reduce AL after esophagectomy, but no studies have yet demonstrated associations between preoperative improvement of inflammation-based and/or nutritional markers and reductions in the frequency of AL. Previous studies have revealed that preoperative immunonutritional treatment might improve early postoperative nutritional status and reduce postoperative complications in gastrointestinal cancer surgery [28]. Although a recent systematic review could not confirm any superiority of enteral immunonutrition compared to enteral nutrition in terms of the development of AL after esophagectomy, preoperative immunonutrition was reported to improve postoperative CRP and albumin levels, which might contribute to reduced AL [29]. Further prospective studies are warranted to validate whether immunonutritional treatment as assessed by CAR is associated with reductions in the frequency of AL.

Several limitations to this study should be acknowledged. First, this study was a retrospective study in a single institution. Second, important factors that could potentially affect AL, such as blood flow, tension at the anastomosis and the technical skill of the surgeon, were not controlled for in this study. Third, although we determined cut-off values for inflammation-based and/or nutritional markers based on the results of ROC curve analyses as an objective statistical method, no optimal, standardized method to determine optimal cut-offs has been established. Finally, since the study period for this investigation was long, changes in preoperative treatment strategies and operative procedures might have affected the results. Further prospective studies with larger numbers of patients are needed to validate the utility of preoperative CAR to predict AL of cervical anastomosis after esophagectomy for esophageal cancer.

## Conclusion

High preoperative CAR (≥ 0.0139) provided an independent predictor of AL after esophagectomy for thoracic esophageal squamous cell carcinoma. Although the cause of AL is considered to be multifactorial, preoperative CAR may be a useful indicator in preoperative management to reduce AL using methods such as neoadjuvant treatment and nutritional intervention.

## Data Availability

The datasets generated during and/or analyzed during the current study are not publicly available due to hospital regulations.

## Abbreviations

AL: Anastomotic leakage
CRP: C-reactive protein
CAR: C-reactive protein-to-albumin ratio
NCD: National Clinical Database
PNI: Onodera’s prognostic nutritional index
mGPS: Modified Glasgow prognostic score
BMI: Body mass index
CCI: Charlson comorbidity index
ASA-PS: The American Society of Anesthesiologists classification of physical status
MIE: Minimally invasive esophagectomy
HALS: Hand-assisted laparoscopic surgery
CT: Computed tomography
PET: Positron emission tomography
5-FU: 5-Fluorouracil
NAC: Neoadjuvant chemotherapy
ECCG: The Esophagectomy Complications Consensus Group
CDC: Clavien-Dindo classification
NAL: No anastomotic leakage
ROC: Receiver operating characteristic
OR: Odds ratio
CI: Confidence interval
AUC: Area under the curve

## References

[1] Markar S, Gronnier C, Duhamel A, Mabrut JY, Bail JP, Carrere N, Lefevre JH, Brigand C, Vaillant JC, Adham M (2015). The impact of severe anastomotic leak on long-term survival and cancer recurrence after surgical resection for esophageal malignancy. Ann Surg.

[2] Blencowe NS, Strong S, McNair AG, Brookes ST, Crosby T, Griffin SM, Blazeby JM (2012). Reporting of short-term clinical outcomes after esophagectomy: a systematic review. Ann Surg.

[3] Takeuchi H, Miyata H, Ozawa S, Udagawa H, Osugi H, Matsubara H, Konno H, Seto Y, Kitagawa Y (2017). Comparison of short-term outcomes between open and minimally invasive esophagectomy for esophageal cancer using a nationwide database in Japan. Ann Surg Oncol.

[4] Haskins IN, Baginsky M, Amdur RL, Agarwal S (2017). Preoperative hypoalbuminemia is associated with worse outcomes in colon cancer patients. Clin Nutr.

[5] De Magistris L, Paquette B, Orry D, Facy O, Di Giacomo G, Rat P, Binquet C, Ortega-Deballon P (2016). Preoperative inflammation increases the risk of infection after elective colorectal surgery: results from a prospective cohort. Int J Colorectal Dis.

[6] Li P, Wang X, Lai Y, Zhou K, Tang Y, Che G (2019). The prognostic value of pre-treatment prognostic nutritional index in esophageal squamous cell carcinoma: a meta-analysis. Medicine (Baltimore).

[7] Wei XL, Wang FH, Zhang DS, Qiu MZ, Ren C, Jin Y, Zhou YX, Wang DS, He MM, Bai L (2015). A novel inflammation-based prognostic score in esophageal squamous cell carcinoma: the C-reactive protein/albumin ratio. BMC Cancer.

[8] Wu CC, Li SH, Lu HI, Lo CM, Wang YM, Chou SY, Chen YH (2018). Inflammation-based prognostic scores predict the prognosis of locally advanced cervical esophageal squamous cell carcinoma patients receiving curative concurrent chemoradiotherapy: a propensity score-matched analysis. PeerJ.

[9] Oshi M, Kunisaki C, Miyamoto H, Kosaka T, Akiyama H, Endo I (2018). Risk Factors for anastomotic leakage of esophagojejunostomy after laparoscopy-assisted total gastrectomy for gastric cancer. Dig Surg.

[10] Yu Y, Wu Z, Shen Z, Cao Y (2020). Preoperative C-reactive protein-to-albumin ratio predicts anastomotic leak in elderly patients after curative colorectal surgery. Cancer Biomark.

[11] Sakamoto W, Ohki S, Kikuchi T, Okayama H, Fujita S, Endo H, Saito M, Saze Z, Momma T, Kono K (2020). Higher modified Glasgow Prognostic Score and multiple stapler firings for rectal transection are risk factors for anastomotic leakage after low anterior resection in rectal cancer. Fukushima J Med Sci.

[12] Charlson ME, Pompei P, Ales KL, MacKenzie CR (1987). A new method of classifying prognostic comorbidity in longitudinal studies: development and validation. J Chronic Dis.

[13] Rice TW, Ishwaran H, Blackstone EH, Hofstetter WL, Kelsen DP, Apperson-Hansen C (2016). Worldwide Esophageal Cancer Collaboration I: Recommendations for clinical staging (cTNM) of cancer of the esophagus and esophagogastric junction for the 8th edition AJCC/UICC staging manuals. Dis Esophagus.

[14] Low DE, Alderson D, Cecconello I, Chang AC, Darling GE, D'Journo XB, Griffin SM, Holscher AH, Hofstetter WL, Jobe BA (2015). International consensus on standardization of data collection for complications associated with esophagectomy: Esophagectomy Complications Consensus Group (ECCG). Ann Surg.

[15] Clavien PA, Barkun J, de Oliveira ML, Vauthey JN, Dindo D, Schulick RD, de Santibanes E, Pekolj J, Slankamenac K, Bassi C (2009). The Clavien-Dindo classification of surgical complications: five-year experience. Ann Surg.

[16] Toiyama Y, Shimura T, Yasuda H, Fujikawa H, Okita Y, Kobayashi M, Ohi M, Yoshiyama S, Hiro J, Araki T (2016). Clinical burden of C-reactive protein/albumin ratio before curative surgery for patients with gastric cancer. Anticancer Res.

[17] Moghadamyeghaneh Z, Hwang G, Hanna MH, Phelan MJ, Carmichael JC, Mills SD, Pigazzi A, Dolich MO, Stamos MJ (2015). Even modest hypoalbuminemia affects outcomes of colorectal surgery patients. Am J Surg.

[18] Jiang H, Hua R, Sun Y, Guo X, Liu Z, Su Y, Li B, Yang Y, Zhang H, Li Z (2020). Risk factors for anastomotic complications after radical mckeown esophagectomy. Ann Thorac Surg.

[19] Mohri Y, Miki C, Kobayashi M, Okita Y, Inoue M, Uchida K, Tanaka K, Inoue Y, Kusunoki M (2014). Correlation between preoperative systemic inflammation and postoperative infection in patients with gastrointestinal cancer: a multicenter study. Surg Today.

[20] Ge X, Cao Y, Wang H, Ding C, Tian H, Zhang X, Gong J, Zhu W, Li N (2017). Diagnostic accuracy of the postoperative ratio of C-reactive protein to albumin for complications after colorectal surgery. World J Surg Oncol.

[21] Lohsiriwat V, Lohsiriwat D, Boonnuch W, Chinswangwatanakul V, Akaraviputh T, Lert-Akayamanee N (2008). Pre-operative hypoalbuminemia is a major risk factor for postoperative complications following rectal cancer surgery. World J Gastroenterol.

[22] Gibbs J, Cull W, Henderson W, Daley J, Hur K, Khuri SF (1999). Preoperative serum albumin level as a predictor of operative mortality and morbidity: results from the National VA Surgical Risk Study. Arch Surg.

[23] Irvin TT, Hunt TK (1974). Effect of malnutrition on colonic healing. Ann Surg.

[24] Brasken P, Renvall S, Sandberg M (1991). Fibronectin and collagen gene expression in healing experimental colonic anastomoses. Br J Surg.

[25] Gabay C, Kushner I (1999). Acute-phase proteins and other systemic responses to inflammation. N Engl J Med.

[26] Balkwill F, Mantovani A (2001). Inflammation and cancer: back to Virchow?. Lancet.

[27] Sparreboom CL, Wu Z, Dereci A, Boersema GS, Menon AG, Ji J, Kleinrensink GJ, Lange JF (2016). Cytokines as early markers of colorectal anastomotic leakage: a systematic review and meta-analysis. Gastroenterol Res Pract.

[28] Li XK, Zhou H, Xu Y, Cong ZZ, Wu WJ, Luo J, Jiang ZS, Shen Y (2020). Enteral immunonutrition versus enteral nutrition for patients undergoing oesophagectomy: a systematic review and meta-analysis. Interact Cardiovasc Thorac Surg.

[29] Giger U, Buchler M, Farhadi J, Berger D, Husler J, Schneider H, Krahenbuhl S, Krahenbuhl L (2007). Preoperative immunonutrition suppresses perioperative inflammatory response in patients with major abdominal surgery-a randomized controlled pilot study. Ann Surg Oncol.

